# Is mother’s financial autonomy associated with stunting among children aged 7–35 months: An empirical study from India

**DOI:** 10.1371/journal.pgph.0000134

**Published:** 2022-01-05

**Authors:** Shirisha P., Anjali Bansal

**Affiliations:** 1 Department of Humanities & Social Science, Indian Institute of Technology, Madras, India; 2 International Institute of Population Sciences, Mumbai, India; University of Ghana, GHANA

## Abstract

Stunting depicts chronic deprivation and is a huge public health problem in several developing countries. Considering the sociocultural and sociodemographic factors of India, we aimed to examine the relationship between maternal autonomy and stunting among children <35 months. We have used the data from the latest round of National Family health survey conducted in 2015–16. The main exposure variable was women’s autonomy which are represented in our study by the four dimensions- decision-making, physical mobility, financial autonomy, attitudes towards domestic violence, the main predictor variable was stunting among children. Chi-square analysis, univariate and multivariable binary logistic regression analysis were performed to find the association of childhood stunting and women’s autonomy. The results were reported at 5% level of significance. All the autonomy variables have shown a significant association with child stunting at 5% level of significance. The unadjusted odds of stunting were found to be significant with respect to all the four dimensions of autonomy variables except physical autonomy. However, after adjusting for other explanatory factors attenuated these relationships and made them statistically insignificant except for women’s economic autonomy (AOR = 0.91; 95% C.I.-(0.85, 0.98)) which was found to be significantly affecting the child’s status of stunting. Our study reinforces that maternal autonomy is a significant predictor of childhood stunting. Hence, we recommend that policy makers, while designing interventions and policies, must address the socioeconomic inequalities at the community level while devising ways to improve women’s empowerment. As it has far-reaching consequences on the nutrition status of the upcoming generations.

## Introduction

According to the World health organization (WHO), Low birth weight (LBW) and malnutrition emerged as a leading cause of death among under-5 children, which translates to approximately 15,000 deaths per day [1]. Moreover, there are economic ramifications of malnutrition as well [2]. Stunting or low height-for-age is a chronic form of malnutrition implying long spells of nutrition deprivation. Even after an impressive 20% relative decrease in stunting, still 40.6 million children remain stunted [3]. Occurrence of stunting increases the risks of non-communicable diseases such as hypertension, diabetes, affecting the cognitive and physical development of children [4]. It was also found to be directly associated with the economic outcomes, an increase of 1 cm in height was found to be associated with four percent and six percent increase in wages for men and women respectively [5]. In India, stunting among under 5 children has declined substantially from 52% in NFHS-1 (1992–93) to 38% in NFHS-4 (2015–16) [6,7].

According to the United Nations International children’s emergency fund’s (UNICEF) conceptual framework, underlying factors, such as feeding and care practices, maternal autonomy, household food security and community health services directly affect dietary intake, morbidity and child nutritional status [8,9]. There is ample empirical evidence showing that characteristics of mothers like education, health, domestic violence faced by mothers, etc. have a significant impact on a child’s nutrition status [10–12]. The characteristics of caregivers are also found to be associated with the nutrition status of children, the care givers characteristics and socioeconomic factors explain 54% of variance in children’s height-for-age [13]. As women are primary caregivers who have the responsibility of providing care to the children, mother’s degree of autonomy at the household level is pivotal to child’s nutrition as it may enhance her ability to spend in interest of her children or may impede her ability to do so [8,14–17]. This could be a plausible pathway through which maternal autonomy affects a child’s nutritional status.

Cultural and economic factors such as the ownership of the resource, control and its utilization, legal and ideological structures, education, information determine the women’s position and status in a community, also decision making autonomy in household expenditure and health care seeking behavior also reflects her position in a household [18]. It is a well-established fact now that households do not work necessarily in a unitary fashion but men and women allocate food and non-food resources differently, based on their bargaining power [15,19,20]. The control of women over income positively influences household food security and nutrition, as they are likely to spend higher on nutrition inputs such as food when compared to men [15,19,21–23]. This could make a difference to the child’s nutrition outcome, for instance in Dominican Republic the children of low-income female-headed households achieved superior nutrition status than male-headed households of same income [24]. Women’s autonomy was found to be associated with health and welfare of children and households [25,26].

The concept of women’s autonomy is multidimensional and there is no uniformity in its definition and measurements [15,26–29]. Several studies in the past have used only a single component for autonomy [17,30–32]. Nevertheless, as autonomy is a multidimensional concept, studying a single component of autonomy could dilute the effect of other important components. Recent literature in India and the rest of the world has included many indicators of autonomy capturing different aspects which could be significantly associated with health behaviors/outcomes [31,33–39]. For instance, a study conducted to examine the role of women’s empowerment in explaining the nutrition status of the children among the rural and tribal women of Karnataka. The measures of autonomy in the study were—physical mobility within and outside the village, domestic violence, position in the household and involvement in decision-making, income and employment status [39]. Another study defines autonomy based on four components- financial, decision-making, physical mobility, domestic violence [40].

Our study aims to contribute to the existing knowledge on the association between women’s autonomy and development outcomes in the context of India and developing countries in general. Drawing upon the latest nationally representative household datasets for India, for children under three years of age with special focus on the mother’s autonomy [41]. Stunting is an indicator of chronic malnutrition, and is therefore liable to be influenced by a mother’s past decision-making autonomy like whether she wanted pregnancy, or duration of breastfeeding etc. But, the degree of autonomy may be varying with time and hence duration of mismatch is likely to increase more if the sample includes older children (>2 years) [42]. Hence, we have restricted the children’s age to not more than 35 months.Although, earlier attempts have been made to understand how autonomy affects children’s health outcomes [25,36,37,40,43]. However, the studies are limited to either one of the dimensions of autonomy or to a specific geographical region of India.

### Data & method

As the concept of autonomy is multidimensional and has no standard definition, different studies have defined autonomy differently and have measured different aspects of it. For instance, certain studies have drawn from the qualitative research preceding it, while some use the components from the surveys. (Shroff et al., 2011) measured autonomy with the seven components, ‘dimensions in which women make decisions and control resources within the family’, expanding the definition of autonomy to include more decision-making dimensions like ‘household decision-making autonomy, child-related decision-making autonomy, (financial autonomy), (mobility autonomy), freedom of movement (mobility), acceptance of domestic violence, and experience of domestic violence’. Chakraborty and Anderson (2011) constructed an autonomy scale using Principal Component Analysis based on responses of women on final say on their own healthcare, final say on making large household purchases, final say on making purchases for daily needs, and final say on visits to family or relatives [44]. Kamiya et al. (2018) has included five components keeping in mind the multidimensionality -autonomy: (i) self-efficacy and (ii) self-esteem as psychological aspects, (iii) decision-making power as a familial/interpersonal aspect, (iv) freedom of mobility as a sociocultural aspect, and (v) control of money as an economic aspect [35,45].

The present study used data from the latest round of National Family Health Survey (NFHS), conducted in 2015–2016. NFHS is Indian equivalent of Demographic and health survey (DHS) and is one of the principal sources of data on fertility, family planning, maternal and child health, nutrition, mortality, environmental health, HIV, malaria as well as provision of health services. Hence, respondents include men, women and children of different age categories. Around 601,509 households, 699,686 women aged 15–49 years, 112,122 men aged 15–54 years, and 259,627 children aged 0–59 months were interviewed. The survey also categorized respondents based on their, majority of the respondents were Hindus (81%), 14% Muslims, 2% Christians and Sikhs each while Buddhist/neo-Buddhist were only 1%. The households are again categorized based on the socially constructed category of caste- Scheduled castes (SC), Scheduled tribes (ST), Other Backward castes (OBCs), and others (non-SC/ST/OBCs). Background characteristics such as age, sex, religion, castes etc. are likely to influence women’s behavior and hence they are included in the survey [6]. The survey provides state, and national level estimates of demographic and health parameters as well as data on various socio-economic and program dimensions, which are critical for implementing the desired changes in demographic and health parameters. A two-stage stratified sampling was conducted in 29 states and seven union territories (for detailed sampling see ref, no.6). The survey for the first time in 2015–16, provided district-level estimates on the various key indicators associated with the demographic and health parameters for the country. NFHS-4 interviewed 601,509 households, 699,686 women aged 15–49 years, 112,122 men aged 15–54 years, and 259,627 children aged 0–59 months. Since the objective was to establish linkages between stunting status of children and women autonomy, so the birth recode NFHS data was used for the analysis where 259,627 children aged 0–59 months were interviewed. We have selected the sample of children under 3 years (7–35 months children), as the early malnutrition status of children has serious implications in the future [34]. NFHS-4 in their schedule collected information from two approaches, firstly district module which provides estimates at district level with a shorter version of questionnaire and second at state module with a longer version but only alternate selected households of 30% selected primary sampling units (PSU). So, the information of the autonomy related variables comes from the women’s schedule in which only a subsample from a PSU was selected [6]. The final sample selected for the analysis included 17,439 children aged 7–35 months.

### Nutritional status of young children

Dataset uses WHO Child Growth Standards as the reference population; and the indices includes z-scores of standard deviation (SD) units from the median of the reference population. The height-for-age z score (HAZ) reflects linear growth and chronic undernutrition in early childhood (stunting) as mentioned earlier. Stunting is represented as − 2 standard deviations from the reference population median representing the cut-off point of HAZ scores. Children having z-scores greater than − 2.00 were coded 0, stands for- not stunted/underweight while children having z-scores of less or equal to − 2.00 were coded as stunted/underweight, that is 1 [46].

### Predictors to determine the nutritional status of young children

We have included the predictors which were included in previous studies on women’s autonomy and child nutrition [16,31,33,47]. The following household characteristics were explored from previous studies and included—place of residence (urban, rural), religion (Hindu, Muslim, Christian, others), caste of household- Schedule Caste (SC), Schedule Tribe (ST), Other backward caste (OBC), Others), geographic region (North, Central, East, North east, West, South), wealth quintile (Poorest, poorer, middle, richer, richest), mother’s characteristics which included age of mother (15–24, 25–34, 35–49), her educational Status (No education, primary, secondary higher), working status of mother (non-working, working), sex composition of child (boy, girl), age of child (7–11, 12–23, 24–25 months), birth order of children (1, 2,3, more than 3), and maternal autonomy variables.

We measured maternal autonomy through various indicators of women empowerment representing measures of different dimensions at individual, household and aggregate level, and at the household level the household- decision-making and mobility [42].

### Decision-making autonomy component includes decision on

1. Own healthcare
2. Purchase of large household purchases
3. Visiting their family or relatives

A women’s response against each decision-making variable is summed up, the summed-up score varied from 0–3. Then a binary variable was created where ‘0’ denotes that women has no participation in decision in any of the variables, and 1 denotes lower level of decision-making autonomy.

### Physical autonomy component includes

1. Is allowed to go alone to the market.
2. Is allowed to go alone to the health clinic.
3. Is allowed to go alone to places outside the community.

A women’s response against each physical autonomy variable is summed up, the summed-up score varies from 0–3. Then a binary variable was created where ‘0’ denotes that women have no physical autonomy, and 1 denotes lower level of mobility among women.

### Financial autonomy component includes

1. Knowledge of loans given to women to start or expand a business of their own
2. Has bank account of her
3. Has money of her own that she can decide how to spend.

A women’s response against each economic autonomy variable is summed up, the summed-up score varied from 0–3. Then a binary variable was created where ‘0’ denotes that women have no economic autonomy, and 1 denotes that women have a lower level of financial autonomy.

### Attitude towards domestic violence includes if husband is justified in beating her

1. She refuses to have sex with him
2. She Argue with Him
3. Food not cooked properly
4. She go out without telling him
5. She Neglects the children

A lower score indicates better self-esteem of women and higher status of women in the household. A women’s response against each variable of domestic violence was summed up, the total score varied from 0–5. If a woman does not justify beating in all the five scenarios, she was given a code 0 indicating high autonomy, and 1 if the woman justifies beating in any one of the scenarios.

### Statistical analysis

Descriptive analysis, univariate and multivariate logistic regression analysis were used to determine the factors affecting the nutritional status among children. Initially univariate analysis (unadjusted logistic analysis) was performed, the variables which were found to be significant (p<0.05) were included in the multivariate model. We used the binary logistic regression analysis as it models the log of the odds of an outcome occurring in terms of a vector of independent variables. The model in the study is defined as:

$$\log\;(Y)=a+b_{1}X_{1}+b_{2}X_{2}$$

Where log (Y) is the natural logarithm of the odds of the outcome(stunting status of children, binary variable), ’a’ is the intercept and b_1_; b_2_ are the coefficients associated with each predictive variable, The standard errors for all the logistic regression were adjusted for all the clusters. The details and methodology of the adjustment of clusters is available elsewhere [48]. The *svyset* command in Stata was used to adjust the standard errors for all the clusters.

For selection of predictors to be fitted in the final model we have used Lasso is an acronym for “least absolute shrinkage and selection operator and used as for prediction, model selection and to draw inferences. It does so by imposing a constraint on the model parameters such that regression coefficients for some variables shrink toward zero. Therefore, for those variables are eliminated whose regression coefficient equal to zero after the shrinkage. While, those with non-zero regression coefficients are most strongly associated with the response variable and hence are retained. The double selection lasso method has been used for model selection i.e. to select potential control variables to be included in the model and is more rigorous approach. It adjust for complex survey design characteristics such as sampling weight. The model handles high dimensional data (large number of independent variables) and correctly estimates the standard error of interest. A total of three models were estimated- Model 1, regression analysis was run for children aged 7–35 months to assess the associations of their stunting status with the maternal autonomy (which were significant in univariate regression analysis). Model 2 –was an adjusted model assessing the stunting status for children aged 7–35 months controlling for all significant household characteristics and maternal autonomy and Model 3—assessing the stunting status controlling for all significant independent variables that were significant in univariate analysis. The analyses were conducted using Stata version 15, and all the results were reported at a 5% level of significance [49].

### Ethics statement

NFHS 4 survey has been conducted by International Institute of Population Sciences (IIPS) under the aegis of Ministry of Health and Family Welfare (MoHFW). The approval for NFHS-4 was obtained from the ethics review board of the International Institute for Population Sciences (IIPS), Mumbai, India. ICF International Review Board (IRB) have reviewed and approved the survey. A written informed consent was obtained from the participants for participation in this survey before commencement of the interview. NFHS-4 is an anonymous publicly available dataset with no identifiable information of the survey participants and accessible upon a granted request from the Demographic and Health Surveys (DHS) Program at https://dhsprogram.com/data/available-datasets.cfm. Therefore, no ethics statement is required for this work.

## Results

This section reports the results, first the descriptive statistics of children aged 7–35 months, second the bivariate analysis between the stunting status and household, mothers’ characteristics and maternal autonomy variables, and third of the determinants of child stunting outcomes.

### Sample characteristics

**Table 1** shows the prevalence of stunting and different components of maternal autonomy at 95% CI. The prevalence of physical autonomy is the highest amongst all the maternal autonomy components.

**Table 1:** Prevalence along with 95% CI.

Variables	Prevalence (95% CI)
Stunting	39.15 (38.94–39.35)
**Maternal Autonomy**	
Physical Autonomy	94.08 (93.86–94.32)
Financial Autonomy	71.07 (70.62–71.54)
Decision Making	81.49 (81.10–81.89)
Attitude towards domestic violence	42.23 (41.74–42.74)

**Table 2** reported the characteristics of the sampled children aged 7–35 months. Among the 17,349 children aged 7–35 months, with the majority in the age group of 12–35 months (39%) were stunted. Almost an equal sex distribution is noticed with 49% females and 51% males in the sample. Around 60% of children had either one or more siblings. Majority of children of the sample population were Hindus (78%) and resided in the rural areas (71%), with only a quarter belonging to categories other than SC/ST or OBC category. Around 45% of children belonged to households categorized to have poor socio-economic standards of living. Though more than half (52%) of the women belonged to the age group of 25–34 years, still a considerable proportion (40%) were 15–24 years. A quarter of the women in the sample population did not have any level of education and the majority of them were (around 85%) not working at the time of interview. About 80% of the mothers have reported involvement in household decision-making. 92% reported autonomy in physical mobility, while 73% had mothers reported minimal financial autonomy, and 55% had mothers not justifying the domestic violence attitude towards them.

**Table 2:** Descriptive statistics of the cohort, NFHS-4, 2015–16 (weighted frequency).

Household characteristics	N	Percentage
**Place of residence**		
Urban	5,057	29.00
Rural	12,382	71.00
**Religion**		
Hindu	13,614	78.07
Muslim	2,990	17.15
Christian	411	2.36
Others	423	2.43
**Caste**		
SC	3,525	20.22
ST	1,783	10.23
OBC	7,902	45.31
Others	4,228	24.25
**Region**		
North	2,337	13.40
Central	4,392	25.19
East	4,254	24.39
North east	572	3.28
West	2,381	13.65
South	3,502	20.08
**Wealth quintile**		
Poorest	3,979	22.82
Poorer	3,785	21.70
Middle	3,575	20.50
Richer	3,260	18.69
Richest	2,841	16.29
**Mothers characteristics**		
**Educational status of mother**		
No education	4,597	26.36
Primary	2,214	12.70
Secondary	8,486	48.66
Higher	2,141	12.28
**Age of mother**		
15–24	7,086	40.63
25–34	9,225	52.90
35–49	1,129	6.47
**Working status**		
Non-working	14,851	85.16
Working	2,588	14.84
**Sex composition of children**		
Boy	8,883	50.94
Girl	8,556	49.06
**Age of child (in months)**		
7–11	2,502	14.35
12–23	7,594	43.54
24–35	7,343	42.11
**Birth order**		
1	6,828	39.15
2	5,645	32.37
3	2,663	15.27
More than 3	2,304	13.21
**Maternal Autonomy**		
Physical Autonomy		
No	1,362	7.81
Yes	16,077	92.19
**Financial Autonomy**		
No	4,756	27.27
Yes	12,683	72.73
**Decision Making**		
No	3,500	20.07
Yes	13,940	79.93
**Attitude towards domestic violence**		
Not Justified in all	9,516	54.57
Justified in any reason	7,923	45.43
Total	**17,439**	

**Table 3** represents the variation in unadjusted proportion of stunting by different dimensions of women’s autonomy. Chi-square test was performed to study the association between women’s autonomy and child’s stunting status. Around 39% of the children aged 7–35 months were found to be stunted. We found significant association between autonomy variables and children’s stunting at 5% level of significance. In the majority of the households, women had a say for at least one of the three variables representing decision-making autonomy. There was no statistical difference in the proportion of stunted children across the levels of autonomy categories of the three variables of decision-making autonomy. The difference in the proportion of stunting among children whose mothers had decision making autonomy(42%) versus those who did not (38%) was not huge. Similarly, proportion of stunting was higher in children of households where women have no physical autonomy vis a vis where women have high physical autonomy. Stunting and all the domains of financial autonomy are significantly associated. Distribution of women among domains of financial autonomy is relatively even, especially for the domain where the respondent *decides alone to use her money*. There was a statistically significant difference between proportions of stunted between the autonomy levels of each variable of financial autonomy. Proportion of stunting was higher (41%) among children whose mother justifies any form of violence on them.

**Table 3:** Bivariate analysis of autonomy variables by stunting in India, NFHS‐4, 2015–16.

Autonomy	Categories	N	Percentage stunted	Chi-square
**Decision Making Autonomy**	** **	** **	** **	** **
Health Care for Yourself	Respondent doesn’t has a say	4,722	41.46	0.001
	Respondent has a say	2,130	38.38	
Large household purchases	Respondent doesn’t has a say	4,516	41.40	0.001
	Respondent has a say	2,336	38.28	
Visit to family and Friends	Respondent doesn’t has a say	4,588	42.00	0.001
	Respondent has a say	2,263	38.07	
Total	Respondent doesn’t has a say in at least one of the above decision	1,474	38.58	0.001
	Respondent has a say in any of the above decision	5,378	42.11	
**Physical Autonomy**				
Allowed to go alone to the market.	Respondent doesn’t has a say	974	40.75	0.004
	Respondent has a say	5,877	39.04	
Allowed to go alone to the health clinic.	Respondent doesn’t has a say	674	41.20	0.004
	Respondent has a say	6,177	39.07	
Allowed to go alone to places outside the community.	Respondent doesn’t has a say	944	40.93	0.020
	Respondent has a say	5,908	39.02	
Total	Respondent doesn’t has a say in movement in any of the above	558	41.01	0.046
	Respondent has a say in at least one of the above of movement	6,293	39.14	
**Economic Autonomy**				
Knowledge of loans given to women to start or expand a business of their own	No knowledge	4,559	41.90	0.001
	Knowledge	2,293	34.92	
Has bank account	No account	3,888	42.79	0.001
	Has a account	2,963	35.46	
Has money of her own that she can decide how to spend.	No	4,473	41.33	0.001
	Yes	2,378	35.90	
Total	No autonomy in all the above	2,172	45.68	0.001
	Autonomy in at least one of the above	4,679	36.89	
**Attitude towards domestic violence**				
husband is justified in beating her if she refuses to have sex with him	Not justified	5,762	38.49	0.001
	Justified	1,090	43.98	
husband is justified in beating her if she Argue with Him	Not justified	4,654	38.14	0.001
	Justified	2,197	41.91	
husband is justified in beating her if the Food not cooked properly	Not justified	5,301	38.02	0.001
	Justified	1,551	44.28	
husband is justified in beating her if she Go out without telling him	Not justified	4,912	38.52	0.008
	Justified	1,939	41.30	
husband is justified in beating her if she Neglects the children	Not justified	4,485	38.51	0.011
	Justified	2,366	40.80	
Total	Not justified all of the reason stated above	3,594	37.76	0.001
	Justified any of the reason	3,258	41.12	
**Total**	** **	**6,851**	**39.29**	** **

Association between the socio-demographic characteristics and stunting status are presented in **Table 4**. All the factors were found to be significantly associated with stunting. Relatively lesser proportion of children were stunted in socioeconomically well-off households (21%) and urban areas (30%). Across religions, higher number of Hindu (40%) and Muslim children (40%) were stunted than Christian and other religions. Highest proportion of stunted children belonged to the ST category (46%) and geographically resided in central India (47%) followed by eastern India (43%) and western India (37%).

**Table 4:** Bivariate analysis of background variables by stunting in India, NFHS‐4, 2015–16.

Household characteristics	Percentage stunted	Chi-square
**Place of residence**		
Urban	29.76	0.001
Rural	43.18	
**Religion**		
Hindu	39.74	0.001
Muslim	39.77	
Christian	25.19	
Others	35.08	
**Caste**		
SC	46.24	0.001
ST	47.57	
OBC	38.52	
Others	31.42	
**Region**		
North	34.47	0.001
Central	47.72	
East	42.64	
North east	34.25	
West	37.29	
South	30.04	
**Wealth quintile**		
Poorest	55.02	0.001
Poorer	45.75	
Middle	36.79	
Richer	30.75	
Richest	21.56	
**Mother’s characteristics**		
**Educational status of mother**		
No education	53.55	0.001
Primary	44.52	
Secondary	35.08	
Higher	19.93	
**Age of mother**		
15–24	39.77	0.001
25–34	38.36	
35–49	43.83	
**Working status**		
Non-working	38.72	0.001
Working	42.53	
**Sex composition of children**		
Boy	41.08	0.001
Girl	37.42	
**Age of child (in months)**		
7–11	23.22	0.001
12–23	42.16	
24–35	41.80	
**Birth order**		
1	33.96	0.001
2	38.62	
3	43.99	
More than 3	51.26	
**Total**	**6,851**	

Frequencies are weighted.

Slightly higher proportion of boys (41%) were stunted than girls (37%). Also, the prevalence of stunting was almost double in the age group of 12–35 months (42%) when compared to less than 12 months (23%) age group. Proportion of children stunted increased with birth order, with 51% of children in the birth order of 3 or more were stunted while 33% of those stunted were first birth order. Higher percentage of children whose mothers were aged between 35–49 years (43%) had no education (53%) were found to be stunted than those whose mothers aged between 25–34 years (38%) and had completed secondary (35%) or higher secondary (19%) education.

**Table 5** presents the adjusted odds with 95% Confidence interval for all the predictor variables of stunting among the children aged 7–35 months in India. Only those variables found to be significant in the univariate analysis were included in the multivariate analysis. In unadjusted odds of stunting with autonomy variables, the children of those women who report economic autonomy, were less likely to be stunted than those who had no autonomy [OR = 0.74, 95% CI- (0.69, 0.79)]. With respect to autonomy in decision-making, children whose mothers possessed decision-making power were less likely to be stunted than those with no decision-making [OR = 0.85, 95% CI- (0.78, 0.91)]. Children whose mothers justified domestic violence, had 1.13 times higher odds than those who did not [95% CI- (1.06, 1.21)]. Physical autonomy was found to be insignificant in the univariate analysis and therefore not included in further models.

**Table 5:** Unadjusted and adjusted odds ratios with 95% Confidence interval (CI) for the predictors of stunting in children aged 7–35 months, India, NFHS‐4, 2015–16.

	Lasso regression for univariate analysis	Model 1	Model 2	Model 3
Physical Autonomy		AOR (95% CI)	AOR (95% CI)	AOR (95% CI)
No autonomy (Ref.)		** **	** **	
Autonomy	1.78 (0.98–1.86)	** **	** **	
**Economic Autonomy**				
No autonomy (Ref.)		1.00	1.00	1.00
Autonomy	0.93***(0.88–0.97)	0.75*(0.7–0.8)	0.87*(0.82–0.94)	0.91*(0.85- 0.98)
**Decision Making Autonomy**				
No autonomy (Ref.)		1.00	1.00	1.00
Autonomy	0.95***(0.9–1.00)	0.89*(0.82–0.96)	0.93 (0.86–1.01)	0.96 (0.88–1.04)
**Attitude towards domestic violence**				
Not justified (Ref.)		1.00	1.00	1.00
Justified	1.03***(1.08–1.56)	1.12*(1.05–1.19)	1.02 (0.96–1.09)	1.00 (0.93–1.07)
**Household Characteristics**				
**Place of residence**				
Urban (Ref.)			1.00	1.00
Rural	0.98 (0.93–1.04)		0.99 (0.91–1.08)	1.00 (0.91–1.09)
**Religion**				
Hindu (Ref.)			1.00	1.00
Muslim	1.16***(1.09–1.23)		1.21*(1.11–1.32)	1.12 (1.02–1.24)
Christian	1.07 (0.96–1.20)		1.11 (0.94–1.3)	1.08 (0.91–1.27)
Others	0.96 (0.85–1.08)		0.94 (0.79–1.13)	0.94 (0.78–1.12)
**Caste**				
SC(Ref.)			1.00	1.00
ST	0.85***(0.79–0.92)		0.85*(0.76–0.95)	0.86*(0.77–0.96)
OBC	0.86***(0.81–0.92)		0.84*(0.77–0.92)	0.85*(0.78–0.93)
Others	0.70***(0.65–0.75)		0.66*(0.59–0.73)	0.69*(0.62–0.77)
**Region**				
North (Ref.)			1.00	1.00
Central	1.32***(1.24–1.42)		1.37*(1.24–1.5)	1.38*(1.25–1.54)
East	1.06 (0.98–1.14)		1.04 (0.93–1.15)	1.08 (0.97–1.21)
North East	0.80***(0.73–0.88)		0.75*(0.65–0.85)	0.80*(0.69–0.92)
West	1.32***(1.2–1.44)		1.27*(1.11–1.44)	1.29*(1.13–1.48)
South	0.91***(0.83–0.99)		0.92 (0.81–1.04)	1.00 (0.88–1.14)
**Wealth Quintile**				
Poorest (Ref.)			1.00	1.00
Poorer	0.80***(0.75–0.85)		0.75*(0.68–0.81)	0.81*(0.74–0.89)
Middle	0.58***(0.55–0.62)		0.55*(0.5–0.6)	0.64*(0.57–0.71)
Richer	0.43***(0.4–0.46)		0.40*(0.36–0.45)	0.50*(0.44–0.57)
Richest	0.32***(0.29–0.35)		0.30*(0.26–0.34)	0.43*(0.37–0.5)
**Women Characteristics**				
**Educational Status**				
No education (Ref.)				1.00
Primary	0.90***(0.84–0.97)			0.90 (0.81–1.00)
Secondary	0.75***(0.7–0.79)			0.77*(0.71–0.84)
Higher	0.52***(0.47–0.58)			0.54*(0.47–0.63)
**Age of women**				
15-24(Ref.)				1.00
25–34	0.90***(0.85–0.95)			0.86*(0.8–0.93)
35–49	0.90***(0.82–0.98)			0.83*(0.72–0.95)
**Working Status**				
Non-working (Ref.)				1.00
Working	1.05***(1.02–1.09)			1.05 (0.96–1.15)
**Sex composition**				
Boy (Ref.)				1.00
Female	0.92***(0.89–0.96)			0.83*(0.78–0.89)
**Age of Child**				
7–11 (Ref.)				1.00
12–23	2.89***(2.65–3.01)			2.78***(2.58–3.00)
24–35	2.85***(2.64–3.10)			2.82***(2.61–3.05)
**Birth Order**				
1(Ref.)				1.00
2	1.11***(1.05–1.17)			1.12*(1.04–1.21)
3	1.28***(1.19–1.37)			1.17*(1.05–1.29)
More than 3	1.36***(1.26–1.46)			

*p<0.05.

Ref.: Reference group; AOR: Adjusted odds ratio; CI: Confidence Interval.

Model 1 of the regression analysis was run to assess the associations of the stunting status for children aged 7–35 months with maternal autonomy variables. It was found that children were found to be less stunted if their mothers have economic autonomy (AOR = 0.75, 95% CI–(0.70, 0.80)). Also, children with mothers who have some autonomy in household decision making were less likely to be stunted than those with no autonomy [AOR = 0.89, 95% CI- (0.82,0.96)]. Those children whose mothers have justified domestic violence were 1.12 times more likely to be stunted [95% CI (1.05, 1.19)] than those who didn’t.

The second model was adjusted to assess the stunting status for children aged 7–35 months controlling for all significant household characteristics and maternal autonomy. Among the maternal autonomy variables, only financial autonomy was found to be significant. When a mother had financial autonomy, then children were 0.87 times less likely to be stunted than those with no autonomy [95% (CI-0.82, 0.94)]. The children belonging to the Muslim religion were more likely to be stunted than Hindu children [(AOR = 1.21, 95% CI-(1.11,1.32)]. Those belonging to the ST category were 0.85 times less likely to be stunted (95% CI- 0.76–0.95)) than those belonging to SC category. The children belonging to Central (AOR = 1.37, 95% CI-(1.24,1.50)), and Western [AOR = 1.27, 95% CI-(1.11,1.44)] part of India, were more likely to be stunted than those in North India [AOR = 0.75, 95% CI-(0.65–0.85)]. Children from the richest quintile are 0.30 times less likely to be stunted (95% CI- (0.26,0.34) than those belonging to the poorest quintile.

The last model (3) assessed the stunting status controlling for all significant independent variables that were significant in univariate analysis. Financial autonomy of mothers is significantly associated with their child’s stunting status, as children whose mothers had financial autonomy were 0.91 times less stunted (95% CI-(0.85,0.98) than those who had no financial autonomy. Mother’s level of schooling was also significantly associated. It was found that children of women who have completed secondary education were 0.77 times less likely to have a stunted child (95% CI-(0.71,0.84). Also, mothers aged between 25–34 years were 0.86 times less likely to have stunted children (95% CI- (0.80,0.93) than those aged younger than 25 years. Female children are less likely to be stunted [AOR = 0.83, 95% CI-(0.78,0.89)] than males. Age of child is a significant covariate as children aged 12–24 months are 2.78 times more likely to be stunted (95% CI-(2.58,3.00)) similar to children aged 24–35 months are than children aged 7–11 months [AOR = 2.82, 95% CI-(2.61,3.05)]. As the number of siblings increases, the chances that all the children in a household are being stunted increases. It was found that when a child has only one sibling, they are 1.12 times more likely to be stunted [95% CI-(1.04,1.21)] than a single child.

## Discussion

The socioeconomic factors were significantly associated with stunting in our study as was in many studies conducted previously in developing countries [50,51]. Children who belonged to poor households, scheduled tribe category or resided in rural areas were more likely to be stunted than their counterparts from wealthy, urban households and other castes. Also, Males were found to be more prone to stunting than females [50,52]. The children whose mothers, aged more than 25 years, had completed secondary or higher level of schooling were less likely to be stunted than those who were younger than 25 years and only at primary level or no education. Our study results corroborate with other study findings [50,53]. The plausible explanation could be that the females, who have not finished secondary education and get pregnant in their teens, are more likely to drop out of school, less likely to be aware about birth control measures. And therefore, pregnancies during the teenage years could result in low birth weight infants who later may develop undernutrition [54].

In our multivariate model, financial autonomy had an independent effect even after controlling for mother’s education and socioeconomic characteristics, which implies that it is an important predictor for explaining stunting. Mother’s power to affect purchasing decisions has been found to be an important predictor related to nutrition and health outcomes of the children. Our study findings with regard to association between financial autonomy and stunting were in congruence with previous studies conducted in relation to children’s health outcomes [35,36,40]. Higher financial autonomy accords higher purchasing power; hence she is better off in negotiations related to household purchases. It influences bargaining power regarding household purchases, allocation of food and childcare and is hence an important predictor of stunting [40,55,56]. An educated mother who has financial independence is more likely to introduce complementary feeding to children at the right age, thus reducing the incidence of stunting [3].

Women’s opinion of the legitimacy of domestic violence has been included as a dimension of autonomy. Similar to our study findings, different studies using opinion or attitudes towards domestic violence as a proxy for autonomy capturing dimensions of domestic violence found it’s effect on stunting to be insignificant [36,40]. The authors explain that the reason for non-significant results could be that the majority of the women in the sample justified domestic violence, implying normalization of the violence [40]. Several previous studies have implied negative impact of domestic violence on child’s nutrition outcome [39,56,57]. The experience of domestic violence may cause psychological, physical or behavioral risks as well as nutritional disorders consequently compromising her ability to take care of the child. The child’s metabolism may be dysregulated in response to stress as well as immunity may be compromised, sequentially leading to impaired cognitive and mental function, reproduction and growth [14,56,57].

The decision-making autonomy has statistically insignificant relation to stunting, similar to the findings by Shroff et al. (2009) [14,25,35,38,40]. Possibly, because the questions asked in NFHS survey provide responses to women’s autonomy more directly related to her own health and nutrition outcomes than her child’s nutrition outcome [25]. The variable was found to be significant in the studies where questions were related to women’s child health care autonomy [31,36,58]. As, most often women are the primary caregivers, their autonomy could considerably influence the child’s growth. As, they may have strong control on decision regarding the factors for child’s health and well-being viz feeding practices, hygiene and health practices, psychological care as well as new born care [14,56]. For example the increased decision-making power was found to be associated with increasing probability of introduction of complementary foods among 6–12 months children in South-Asia, also influencing it’s quality and frequency in other regions [17].

Our results highlight that financial autonomy, of all the dimensions included to measure autonomy, was found to be significantly associated with a child’s nutrition status. In a review of 62 quantitative studies on women’s empowerment and child’s nutrition status, 379 out of 461 associations found with respect to stunting were found to be not significant [42].

There are lessons from a few programmes aimed at improving the overall financial status of households, which empowered women and had a positive impact on her children’s nutrition status as one of the unintended but positive effects of the programme. One such programme is South Asian Poverty Alleviation Programme launched in Andhra Pradesh, which saw positive social impacts on women who were members and their family. Strong and positive impact was seen on children’s nutrition status especially boys whose mother took loan [59].

### Strengths & limitations

The pros of nationally representative data is the huge sample size and the rich data on socioeconomic and demographic characteristics of the household. But, there are certain limitations of our study as we use this dataset. Firstly, the dimensions included to measure autonomy may not be accurately representing the dimension we wish to capture. For instance, the attitude towards domestic violence was not strongly correlated with the experience of domestic violence. Second, this is a cross-sectional study, which facilitates testing of the association and its significance, but it can’t be used to establish causality. Since the data provides growth patterns of the number of children and not the same set of children at different points in time. Hence, future researchers should engage in longitudinal studies with improved study design to establish a causal relationship of the dimension of autonomy and nutrition status. Also, only a handful of studies used Instrumental variables or matching which are statistical manipulation techniques and could help establish better counterfactuals [16,42].

## Conclusion

The findings of our study strengthen the evidence supporting the hypothesis that maternal autonomy is an important predictor of stunting. With high prevalence of stunting, still endangering the lives of millions of under-5 children, the national commitment towards combating all forms of malnutrition has intensified, also the government has launched *Poshan abhiyan*. With the current rate of reduction in stunting, we are unlikely to meet the sustainable development goal (SDG-2) to end hunger, achieve food security and improve nutrition by 2030 especially among young children and the most vulnerable. Therefore, it is imperative that in addition to the investments being made in nutrition specific interventions, policies should also be aimed at improving female’s education and their financial independence. Besides improving the women’s status, it has a far-reaching impact on her child’s nutrition status.

## Abbreviations

GDP: Gross domestic product
HAZ: Height for age z-score
LBW: Low birth weight
NFHS: National Family Health Survey
OBC: Other backward caste
PSU: Primary sampling unit
SC: Scheduled Caste
SD: Standard deviation
SDG: Sustainable development goal
SPPAP: South asian poverty Alleviation programme in Andhra Pradesh
ST: Scheduled Tribes
WAZ: Weight for age z-score
WHO: World health organization
UNICEF: United Nation International Children’s emergency fund
